# Effects of intratracheal administration of nuclear factor-kappaB decoy oligodeoxynucleotides on long-term cigarette smoke-induced lung inflammation and pathology in mice

**DOI:** 10.1186/1465-9921-10-79

**Published:** 2009-08-25

**Authors:** Yu-Tao Li, Bei He, Yu-Zhu Wang, Jing Wang

**Affiliations:** 1 Department of Respiratory Medicine, Peking University Third Hospital of, Beijing, PR China

## Abstract

To determine if nuclear factor-κB (NF-κB) activation may be a key factor in lung inflammation and respiratory dysfunction, we investigated whether NF-κB can be blocked by intratracheal administration of NF-κB decoy oligodeoxynucleotides (ODNs), and whether decoy ODN-mediated NF-κB inhibition can prevent smoke-induced lung inflammation, respiratory dysfunction, and improve pathological alteration in the small airways and lung parenchyma in the long-term smoke-induced mouse model system. We also detected changes in transcriptional factors. In vivo, the transfection efficiency of NF-κB decoy ODNs to alveolar macrophages in BALF was measured by fluorescein isothiocyanate (FITC)-labeled NF-κB decoy ODNs and flow cytometry post intratracheal ODN administration. Pulmonary function was measured by pressure sensors, and pathological changes were assessed using histology and the pathological Mias software. NF-κB and activator protein 1(AP-1) activity was detected by the electrophoretic motility shift assay (EMSA). Mouse cytokine and chemokine pulmonary expression profiles were investigated by enzyme-linked immunosorbent assay (ELISA) in bronchoalveolar lavage fluid (BALF) and lung tissue homogenates, respectively, after repeated exposure to cigarette smoke. After 24 h, the percentage of transfected alveolar macrophages was 30.00 ± 3.30%. Analysis of respiratory function indicated that transfection of NF-κB decoy ODNs significantly impacted peak expiratory flow (PEF), and bronchoalveolar lavage cytology displayed evidence of decreased macrophage infiltration in airways compared to normal saline-treated or scramble NF-κB decoy ODNs smoke exposed mice. NF-κB decoy ODNs inhibited significantly level of macrophage inflammatory protein (MIP) 1α and monocyte chemoattractant protein 1(MCP-1) in lung homogenates compared to normal saline-treated smoke exposed mice. In contrast, these NF-κB decoy ODNs-treated mice showed significant increase in the level of tumor necrosis factor-α(TNF-α) and pro-MMP-9(pro-matrix metalloproteinase-9) in mice BALF. Further measurement revealed administration of NF-κB decoy ODNs did not prevent pathological changes. These findings indicate that NF-κB activation play an important role on the recruitment of macrophages and pulmonary dysfunction in smoke-induced chronic lung inflammation, and with the exception of NF-κB pathway, there might be complex mechanism governing molecular dynamics of pro-inflammatory cytokines expression and structural changes in small airways and pulmonary parenchyma in vivo.

## Introduction

Extensive exposure to cigarette smoke is a principal risk factor associated with chronic obstructive pulmonary disease (COPD). COPD is a complex inflammatory disease involving numerous inflammatory cell types, which have the capacity to release multiple inflammatory mediators. An increase in expression of many of these mediators translates to activation of an inflammatory cascade involving cytokines, chemokines, growth factors, enzymes, receptors, and adhesion molecules [1-4]; specific to COPD are increased levels of tumor necrosis factor-α (TNFα), interferon-γ(IFNγ), interleukin-8(IL-8), macrophage inflammatory protein 1α(MIP-1α), monocyte chemoattractant protein 1(MCP-1), GROα, and matrix metalloproteinase(MMP)-9 [1-4].

NF-κB is a family of critical transcription factors regulating many cytokines, including IL-8, IL-6, TNF-α, GM-CSF, MIP-1, and MCP-1 [5], as well as MMP-9 expression [6]. In the past few years, five mammalian NF-κB family members have been identified and cloned [7-9]. These include NF-κB1 (p50/p105), NF-κB2 (p52/p100), RelA (p65), RelB, and c-Rel. In resting cells NF-κB is retained in the cytoplasm due to inhibitory protein (I-κB) binding. When the cell is appropriately stimulated, I-κB degradation results in the ability of NF-κB to recognition nuclear localization signals of p65, thus it is rapidly transported into the nucleus where it binds to specific κB recognition elements in the promoters of target genes [10]. Chronic exposure to cigarette smoke causes cellular oxidative stress, a key feature in smoking-induced lung inflammation [11-13], and oxidative stress (particularly hydrogen peroxide) can enhance the DNA binding activity of NF-κB [14].

It has been demonstrated in humans and animal models that smoke-induced chronic pulmonary inflammation is associated with increased NF-κB activity in lung cells. Enhanced NF-κB activation has been observed in bronchial biopsies from smokers, macrophages from COPD patients and in guinea pigs exposed to cigarette smoke, with a subsequent increase in IL-8 release [15-17]. During the past few years, tremendous progress has been achieved in our understanding on how intracellular signaling pathways are transmitted in either a linear or a network manner leading to the activation of NF-κB and airway inflammation control [18-20]. However, a detailed role in long-term smoke-induced inflammation and the impact of NF-κB inhibition on histology and airflow obstruction has yet to be determined. Therefore, we have used NF-κB decoy ODNs to block NF-κB activity in mouse lung during long-term smoke exposure. It is well known that transfection of *cis*-element double-stranded oligodeoxynucleotides (decoy) has been identified as a powerful tool in a new class of anti-gene strategies for gene therapy and research [21]. Transfection of decoys corresponding to a specific *cis *sequence results in the attenuation of endogenous *cis*-elements, and subsequent modulation of gene expression [21,22].

We hypothesized that in the long-term smoke-induced mouse model double-stranded ODNs decoy to NF-κB would suppress the pulmonary expression levels of inflammation-related genes and MMP-9/TIMP-1 gene that may play a role in the development of emphysema. The other purpose of this study was to assess the potential of NF-κB decoy ODNs to histological influence. Based on the evidence that early structural changes may occur in peripheral airways of smokers before COPD [23], we further measured small-airway changes. Since NF-κB and AP-1 may regulate each other [24], both of NF-κB and AP-1 activities were measured after intratracheal administration of NF-κB decoy ODNs in 92 day smoke-induced mice. Therefore, the present study was performed to determine the effects of NF-κB decoy ODNs on lung inflammation and pathological changes in the cigarette smoke-induced animal model.

## Materials and methods

### NF-κB decoy ODNs

Double-stranded NF-κB decoy ODNs containing the consensual NF-κB binding site (5'-GGGATTTCCC-3') were generated using equimolar amounts of single-stranded sense and antisense phosphorothioate-modified ODNs (sense strand: 5'-CCT TGA AGG GAT TTC CCT CC-3') as previously described [25]. Briefly, synthetic single-stranded ODNs were dissolved in sterile STE buffer (10 mM Tris, 1 mM EDTA, 100 mM NaCl, pH 8.0), purified by PAGE and quantified by SDS gel electrophoresis (AuGCT company, Beijing, China). Single-stranded ODNs were then annealed for 3 h, during which time the temperature was reduced from 94°C to 25°C. Double-stranded scrambled ODNs were used as negative controls (sense strand: 5'-TTG CCG TAC CTG ACT TAG CC-3') [25]. In the flow cytometry experiment, the sense and antisense NF-κB decoys ODN were modified with FITC labels at both the 5'and 3'end.

### Animals

Male C57/BL6 mice (6–8 weeks of age, 20 ± 0.5 g, Beijing University Animal Center, Beijing, China) were divided into 3 groups, treated for 92 days smoke exposure and started intratracheal instillation on 52 days, administered every 10 days, a total of 4 times: 1 decoy group (n = 8): smoke-exposed followed by intratracheal instillation of NF-κB decoy ODNs (15 nmol in 30 μl of STE buffer/mouse); and negative controls 2 NS group (n = 8): smoke-exposed followed by intratracheal instillation of sterile normal saline (0.9%NS, 30 μl/mouse); and 3 Scr group (n = 8): smoke-exposed followed by intratracheal instillation of scrambled ODNs (15 nmol in 30 μl of STE buffer/mouse). In order to recognize pulmonary function before intratracheal instillation, an additional test has been done, in which 20 mice were divided into 2 groups and treated for 52 days 4 sham group (n = 10): air exposure; 5 smoke-exposed group (n = 10): smoke exposure. These time points were chosen from previous data generated by our group [26,27].

In experiments aimed at NF-κB decoy localization, intratracheal administration to smoke-exposed mice (for 52 days) was performed with FITC-labeled ODNs after 3 h, 24 h, 3 days and 7 days. All animal experimentation was approved by the Local Ethical Committee of Peking University, China.

### Chronic exposure to cigarette smoke

Mice were whole-body exposed to cigarette smoke generated from commercial cigarettes in 300 L inhalation chambers (Derby, USA. Tar = 13 mg, cotinine = 1.2 mg, CO = 15 mg per cigarette). Actual smoke generation method was designed by Masanori Nishikawa, as described previously [16]. The exposure regime consisted of two sessions of 5 cigarettes/hr, interrupted by a 10 min rest period. The exposure regime was conducted twice daily with a minimal four hour interval between sessions, 6 days/week. Carbon monoxide concentration was ranged between10% and 12% after exposure [28], and the mice appeared grossly normal during the entire experimental period. Chamber concentrations of CO were 400–501 ppm (measured by Infrared Gas Analyzer, MODEL GXH-3050A) and particulates (PM10) were 7.88–8.28 mg/m3 (measured by Respirable Aerosol Mass Monitor, MODEL 3511). Animals were maintained on a 12 h light/dark cycle with free access to conventional laboratory food and water. Mice were sacrificed at 24 hour after the last exposure regime.

### Respiratory function

After 52 or 95 days of smoke exposure, mice were anaesthetized by intraperitoneal injection with 1% sodium pentobarbital, and then intubated endotracheally using improved scalp needles. Respiratory function was measured using an Animal Ventilator (Biolab) connected to a pressure sensor. Peak inspiratory flow (PIF) and peak expiratory flow (PEF) were measured, and data were analyzed using Chart 4.1 software.

### Bronchoalveolar lavage cytology, and cytokine assays

On day 95 of the smoke exposure regime, after exsanguination by severing the abdominal aorta, mouse lungs were sequentially lavaged twice with 0.5 ml of Hank's balanced salt solution (HBSS). Recovered aliquots of BALF were pooled. Bronchoalveolar lavage (BAL) cells were pelleted by centrifugation at 1,000 rpm for 8 min. Cell differentials were performed on cytospin preparations stained with Wright-Giemsa, and a total of 200 cells were counted. Supernatant was stored at -80°C. Supernatant TNF-α and IL-6 concentrations were measured using a commercially available ELISA kit (Jingmei Company, Shenzhen, China) according to the manufacturer's specifications. The concentration of pro-MMP-9 was detected in the supernatant of BALF as a commercial kit for MMP-9 was not available [29]. Pro-MMP-9 and TIMP-1 levels were detected using ELISA kits (R&D systems, catalog number: MMP900, MTM100, respectively). The detection limit of TNF-α, IL-6, pro-MMP-9 and TIMP-1 were 7 pg/ml, 4 pg/ml, 3 pg/ml and 1.4 pg/ml, respectively.

### Tissue Processing

Lungs were excised from mice, and the right lobe was tied off, harvested, washed with 4°C PBS solution, weighed and snap-frozen in liquid nitrogen. The left lobe was inflated with 0.25 ml of 4% paraformaldehyde and immersed in fresh 4% paraformaldehyde for 12 h. Tissues were embedded in paraffin and stained with hematoxylin and eosin (H&E).

### Preparation and analysis of lung homogenates for chemokine determination

MCP-1 and MIP-1α concentrations were measured in lung homogenates collected from 95 day smoke-exposed mice. The nitrogen-snap frozen portion of the right lung was cut into small pieces and placed in 4°C PBS solution at 4 ml/g [30], and homogenized on ice (homogenizer: Ingenieurbüro CAT. M. Zipperer GmbH, Germany) for 20 sec at 6,000 rpm three times. Homogenates were centrifuged at 10,000 × *g *at 4°C and stored at -80°C until MCP-1 and MIP-1α levels could be determined by FlowCytomix (BMS8440FF, Bender MedSystems). The limitaton of detection of MCP-1 and MIP-1α concentration were 50 pg/ml and 17 pg/ml, respectively.

### Morphologic and Morphometric Analyses

Intra-alveolar macrophages from H&E stained lung sections in the terminal bronchiole region were counted at 400× magnification by two independent observers in a blind study. Results were expressed as the number of macrophages/mm^2 ^[31].

Quantification of airspace enlargement was determined by mean linear intercept (Lm) ([32-36]) and mean alveolar surface (Am). The measurement of Lm was performed by using a 100×100 μm grid that was randomly positioned in the lung. The length of each grid line, divided by the number of alveolar intercepts, yielded the average distance between alveolated surfaces, or the Lm. The same image was used to measure the Am. An alveolus or airspace is defined as the space surrounded by the alveolar wall, which in the case of an alveolus opening into a duct ends at the mouth of the alveolus. The surface of an airspace cross-section was calculated and divided by the number of alveoli to obtain the Am.

The destruction of alveolar walls was quantified by the destructive index (DI) [32]. Briefly, a grid with 42 hairline crosses was superimposed on the lung field. Structures lying under the cross-points were classified as normal (N) or destroyed (D) alveolar and/or duct spaces. Points falling over other structures, such as duct walls, alveolar walls, etc., were not considered in the calculations. The DI was calculated using the following formula:

### Analysis of small airways fibrosis and inflammation

Masson trichrome stain was used on consecutive tissue sections as a further means to identify fibroblasts and was carried out using Masson trichrome staining Kit (BASO Co., Tai wan) according to the manufacturer's instruction. Lung sections were processed for Masson's trichrome staining to detect collagen and elastin, and analyzed by two separate pathologists in a blinded fashion. Small airway fibrosis and inflammation scores were determined as described before [37].

### Flow cytometry

Localized FITC-labeled NF-κB decoys in macrophages were detected in BALF collected from 52 day smoke-exposed mice after FITC-labeled ODNs or 0.9% NS administration at 24 h, 3 days and 7 days. BALF cells were harvested by sequentially lavaging mouse lungs twice with 0.5 ml of HBSS containing 2 mM EDTA and were assayed for non-vitality by staining with 0.4% trypan blue(Sigma). Then the cells were pelleted by centrifugation at 800 rpm for 8 min, differentiated as described above and filtered through nylon mesh prior to flow cytometry analysis. Cells were incubated (for 30 min on ice in PBS containing 2% Bovine Serum Albumin, 0.1% Sodium azide) with either PE-conjugated anti-mouse F4/80 (Serotec, MCA497PE) or PE-conjugated anti-mouse IgG antibody as a isotype control (BD Pharmingen,553989). Cells were washed, fixed with paraformaldehyde (0.25%), and analyzed using a FACSCalibur (BD Biosciences, San Jose, CA, USA).

### Nuclear Protein Extraction

Fresh snap-frozen mouse lung tissue was weighed, cut into small pieces, and homogenized directly in Cytoplasmic Extraction Reagent I (Pierce, 78833). The mix solution was vortexed vigorously on the highest setting for 15 sec to resuspend the cell pellet, then incubated on ice for 10 min. Ice-cold Cytoplasmic Extraction Reagent II (11 ml) was added to the mix solution, vortexed for 5 sec on the highest setting and incubated on ice for 1 min. The mix solution was centrifuged for 5 min at 16,000 × *g*, and the supernatant was collected in a clean pre-chilled tube.

The nuclei pellet was resuspended on ice in 100 μl of ice-cold Nuclear Extraction Reagent, and vortexed for 15 sec every 10 min for 40 min. The sample tube was centrifuge at 16,000 × *g *for 10 min, and the supernatant (nuclear extract) was collected in a clean pre-chilled tube and stored at -80°C.

### Electrophoretic Motility Shift Assay

Binding reactions were established in 20 μl of binding buffer from the Pierce LightShift Chemiluminescent EMSA Kit (Pierce,20148) using 5 μg of nuclear extract protein per reaction for the consensus probe 5'-biotin labeled: NF-κB 5'-AGT TGA GGG GAC TTT CCC AGG C-3'; AP-1 5'-CGC TTG ATG AGT CAG CCG GAA-3'. Samples were electrophoresed through a 5% polyacrylamide gel for 50 min at 4°C and then transferred to a positively charged nylon membrane for 30 min. DNA was UV cross linked to the membrane, and the membrane was blocked for 15 min by incubation in LightShift Blocking Buffer with gentle shaking. The membrane was then incubated in conjugate/blocking buffer solution for 15 min, washed 4 times for 5 min each in 10 ml LightShift Substrate Equilibration Buffer, followed by incubation in Washing Buffer for 5 min with gentle shaking a total of 4 times. Electrophoretic mobility shifts were visualized using enhanced chemiluminescence solution (Pierce, 20148). The binding bands and probe were analyzed using Kodak software.

### Protein Assay

Protein concentrations in lung homogenates were determined using the bicinchoninic acid (BCA) method.

### Statistical Analysis

All values given represent mean ± standard deviation (STD). Nonparametric Mann-Whitney U-test was used to assess the statistical significance of differences between the groups. Correlations between the BAL analysis data and the MCP-1, MIP-1α levels were assessed with the nonparametric Spearman correlation test. For each analysis, P values less than 0.05 were considered to be statistically significant. Statistical analyses were performed by using the Statistical Package for the Statistical Analysis System 8.1(SAS, Cary, NC, USA).

## Results

### Respiratory function was unaltered after 52 days of smoke exposure

Respiratory function in the smoke-exposed mouse group, as measured with an animal ventilator and connected pressure sensor, was not affected after 52 days of exposure to smoke when compared to sham controls, as illustrated in Table 1.

**Table 1:** Respiratory Function in Cigarette Smoke-Exposed Mice (persistent exposure to smoke for 52 days) and Sham Mice (exposure to air).

Test	Exposure	MEAN ± STD(L/S)	P_r _> Chi-Square
PIF	sham	1.70 ± 0.67	0.7393
	smoke	1.76 ± 0.39	

PEF	sham	7.09 ± 0.39	0.9558
	smoke	7.07 ± 0.24	

The data were expressed as means ± STD and analyzed by using the Mann-Whitney U-test. Statistical significance was accepted at P < 0.05.PIF: peak inspiratory flow; PEF: peak expiratory flow. n = 10–15/group.

### Administration of NF-κB decoy ODNs intratracheally reduced NF-κB activity in the lungs after 92 days smoke exposure

**Figure 1:**
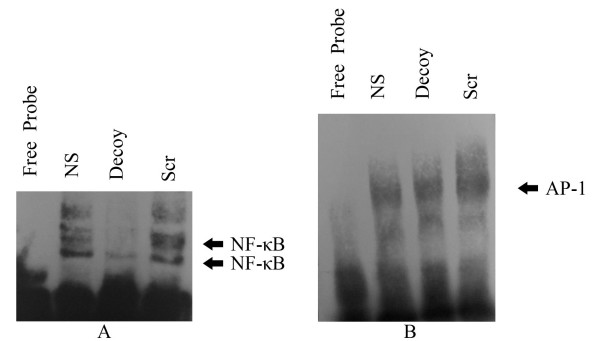
**Demonstration of the impact of local administration of decoy ODNs on NF-κB activation in the lungs of 92 day smoke-exposed mice**. Normal saline (NS), NF-κB decoys ODNs (Decoy) or scrambled ODNs (Scr) were administered by intratracheal instillation on day 52 in smoke-exposed mice. Nuclear protein extracts were prepared from whole lung and assessed for NF-κB DNA-binding activity by electrophoretic mobility shift assay (EMSA). (A) A representative non-autoradiograph of EMSA analysis of level of NF-κB in the nuclear fraction using biotin detection. (B) A representative of the EMSA analysis of level of AP-1 in the nuclear fraction by non-autoradiograph.

The lungs of 92 day smoke-exposed mice were examined for evidence of an NF-κB decoy ODNs-mediated reduction in NF-κB activation in the lungs. Nuclear extracts prepared from whole lung of normal saline (NS) or scrambled ODNs (Scr) intratracheally instillated mice demonstrated strong NF-κB binding activity, as assessed by EMSA (Fig. 1A). As expected, a weak NF-κB-binding activity was observed in whole lung extracts of mice treated with NF-κB decoy ODNs. In contrast, AP-1 binding activity was not significantly changed by NF-κB decoy ODNs administration (Fig. 1B).

### NF-κB decoy ODNs were capable of entry into alveolar macrophages on day 52 of smoke exposure

To show that whether decoy-mediated NF-κB inhibition was sufficient to induce cell non-vitality of BALF cells in 52 day smoke-induced mice, we examined using trypan blue staining at 24 hour after treatment with NF-κB decoy ODNs or normal saline (NS) as a control. The rates of non-vitality cells in BALF were similar to that of NS-treated animals (Table 2). Thus, our results show that treatment of 52 day smoke-induced mice with NF-κB decoy ODNs did not impact on cell survival in BALF.

**Table 2:** Percentage of death cells in the BALF of NS-treated (NS) and NF-κB decoy-treated (Decoy) mice after 24 hours with smoke exposure for 52 days.

Treatment	Non-viable cells %
NS	4.32 ± 3.93
Decoy	5.56 ± 5.53

P_r _> Chi-Square	0.6579

All data are expressed as means ± STD and analyzed by using the Mann-Whitney U-test in two groups of mice. Statistical significance was accepted at P < 0.05. n = 3/group.

**Figure 2:**
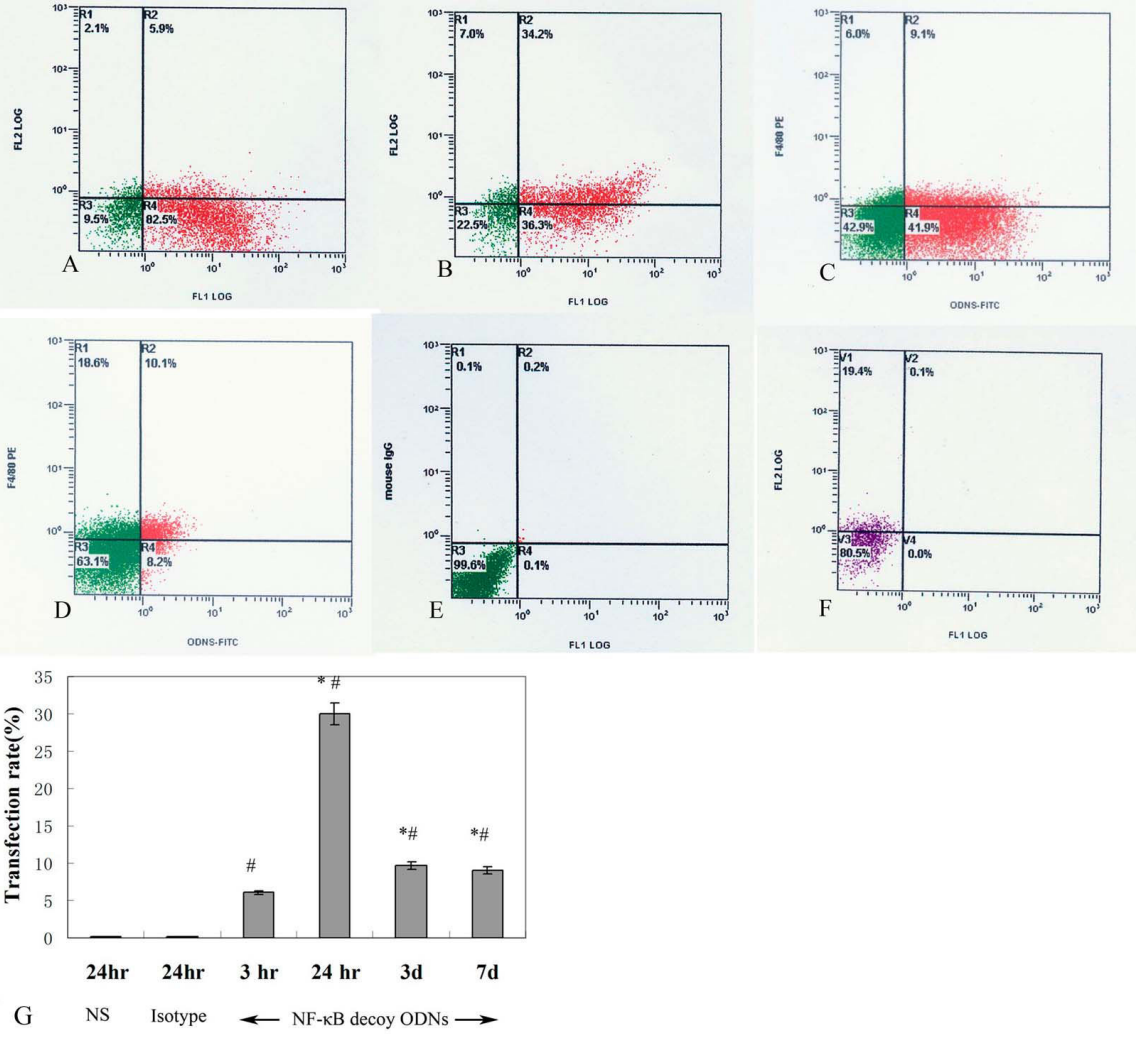
**Dot-plots of FITC-labeled NF-κB decoy ODNs and F4/80 double-positive cells in BALF on day 52 in smoke-exposed mice**. NF-κB decoy ODNs were capable of effective entry into alveolar macrophages in BALF. FITC-labeled NF-κB decoy ODNs were administered intratracheally on day 52 in smoke-exposed mice. As a negative control, smoke exposed mice in 52 days were treated with normal saline (F). After 3 h (A), 24 h (B), 3 days(C) and 7 days (D), macrophages (labeled F4/80) from BALF were assessed for FITC positivity using flow cytometry. A population of FITC-labeled NF-κB decoy ODNs and F4/80 double-positive cells was present in all analysis (R2, higher right quadrant) whereas R1 represented the F4/80-positive, but FITC-ODNs negative macrophages. In BALF, cells collected from mice treated with PE-conjugated isotype IgG antibody (E) as another negative control. Both of the negative controls showed false positive rate (R1+R2+R4) < 5%, which suggested the flow cytometry experiments were not interfered with nonspecific backgrounds. These results were representative of 3 comparable experiments.

To localize NF-κB decoy ODNs *in vivo*, 52 day smoke-exposed mice were administrated FITC-labeled ODNs intratracheally. After 3 h, 24 h, 3 days, and 7 days cells collected from BALF were examined for FITC positivity by flow cytometry; alveolar macrophages were labeled as F4/80. Prior to flow cytometry analysis, cell differential was determined using cytospin preparations stained with Wright-Giemsa, and a total of 200 tabulated cells. We determined that alveolar macrophages in BALF constituted over 95% of total cells (Table 3). In cells collected 24 h after instillation, an observed peak depicted that 30.00 ± 3.30% of the FITC signal was located in macrophages (F4/80)(Fig. 2G and Fig. 2B), which after 7 days persisted at 9.00 ± 0.93% (Fig. 2G and Fig. 2D). Macrophages labeled F4/80 (an transmembrane protein, the best marker for mature macrophages) from BALF were assessed for PE (marked F4/80) and FITC positivity (marked NF-κB decoy ODNs) using flow cytometry. The data analysis was the compilation of quadrant statistics. The co-stained cells (F4/80^+^, FITC-ODNs^+^) were showed by R2 rectangular gating regions. The percentage in the R2 rate indirectly reflected transfection efficiency of NF-κB decoy ODNs to the mature macrophages in vivo.

**Table 3:** Inflammatory cell profile in BALF from NF-κB decoy ODNs (Decoy) and normal saline (NS) treatment in 52 day cigarette smoke-exposed mice.

	Macrophages(%)	Lymphocytes(%)	Neutrophils(%)
Decoy	98.62 ± 0.40	0.60 ± 0.47	0.79 ± 0.56
NS	98.94 ± 1.03	0.59 ± 0.70	0.48 ± 0.38

Pr > Chi-Square	0.7728	0.766	0.3094

All data are expressed as means ± STD. The data were analyzed by using the Mann-Whitney U-test in two groups of mice. Statistical significance was accepted at P < 0.05. n = 4 per/group.

### NF-κB decoy ODNs attenuated macrophage aggregation in smoke-induced chronic inflammation, improved lung function, and reduced MIP-1α and MCP-1 expression

**Figure 3:**
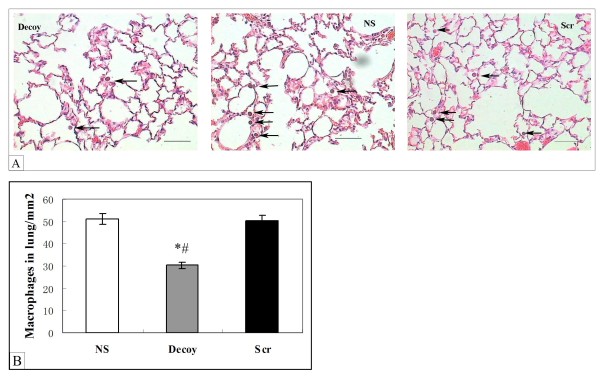
**NF-κB decoy ODNs attenuated macrophage aggregation in smoke-induced chronic inflammation on day 92**. (A) Alveolar macrophages (arrows) are largely observed in the lung parenchyma of smoke-exposed mice on day 92 in both normal saline-treated smoke-induced mice (NS) and scrambled ODNs-treated smoke-induced mice (Scr), but not in NF-κB decoy ODNs administered mice on day 92 of smoke exposure (Original magnification 400×), Bar = 50 μm. (B) Quantitative measurement of intra-alveolar macrophages, expressed as macrophages/mm^2 ^(mean ± STD, n = 8/group). There was clear decrease of macrophage numbers in Decoy mice compared with NS and Scr group. Symbols delineate statistical significance compared to NS mice (*, P < 0.05) and Scr mice (#, P < 0.05). NS: normal saline-treated smoke-induced mice; Decoy: NF-κB decoy ODNs-treated smoke-induced mice; Scr: scrambled ODNs-treated smoke-induced mice.

To demonstrate that the impact of NF-κB decoy ODNs on smoke-induced chronic inflammation, a series of experiments were performed. We analyzed whether intratracheal delivery of NF-κB decoy ODNs could affect smoke-induced macrophage influx, some macrophage-related chemokines and pro-inflammatory cytokines expression, lung function, and cell number in BALF. After smoke exposure for 92 days, macrophage accumulation in the alveolar space was observed in normal saline (NS) and scrambled ODNs (Scr) mice. Treatment with NF-κB decoy ODNs resulted in a reduction in alveolar macrophage accumulation in the alveoli (Fig. 3A). The number of macrophages is tabulated in Fig 3B.

**Figure 4:**
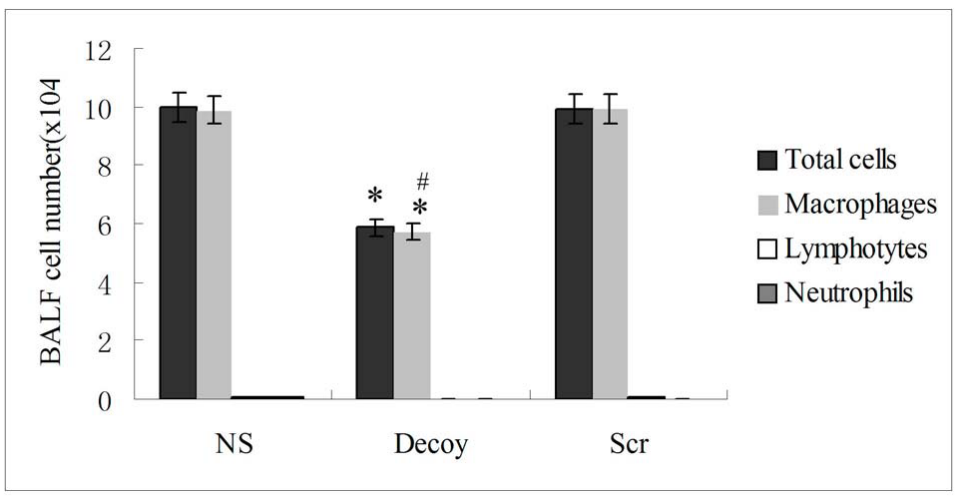
**Treatment with NF-κB decoy ODNs markedly attenuated the number of airway inflammatory cells in smoke-induced chronic airway inflammation on day 92**. Total and differential cell counts were performed on the collected BALF. Data were expressed as mean ± STD (n = 8/group). Symbols delineate statistical significance compared to NS mice (*, P < 0.05) and Scr mice (#, P < 0.05). NS: normal saline-treated smoke-induced mice; Decoy: NF-κB decoy ODNs-treated smoke-induced mice; Scr: scrambled ODNs-treated smoke-induced mice.

**Figure 5:**
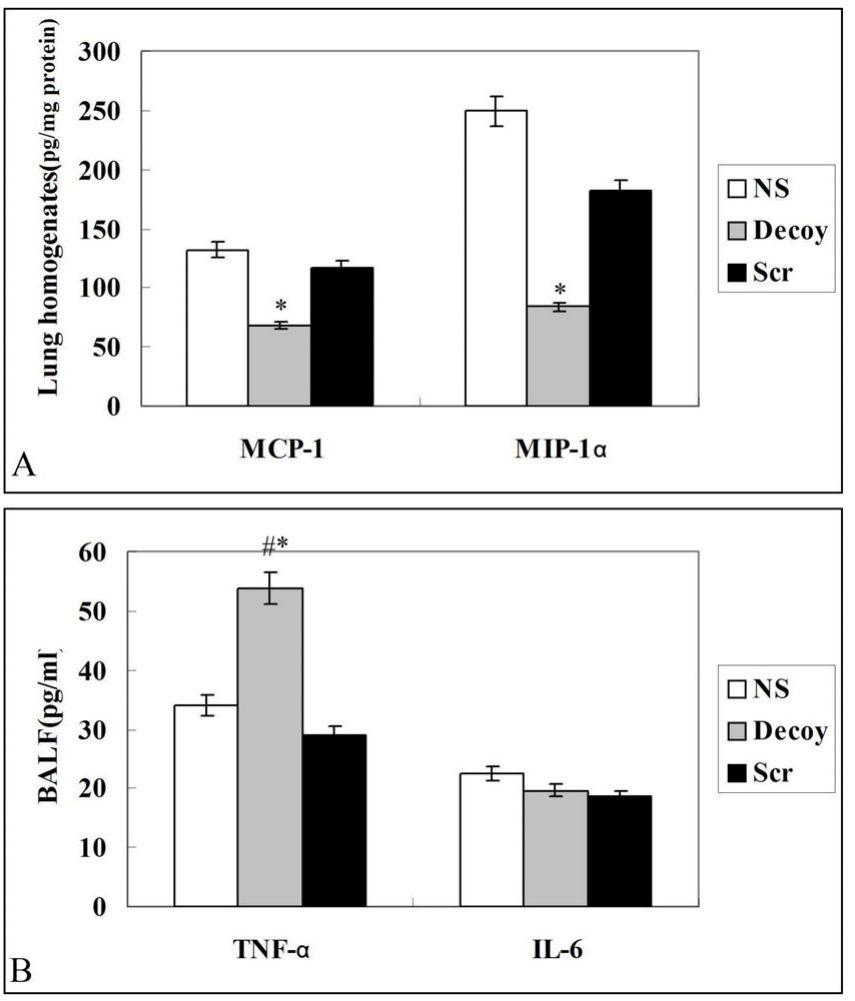
**Lung inflammation at 92 days smoke-exposure after treatment with normal saline (NS), NF-κB decoy ODNs (Decoy) or scrambled ODNs (Scr)**. NF-κB decoy ODNs inhibited MIP-1α and MCP-1 but not IL-6. The level of TNF-α in BALF was increased in Decoy group, compared with the level of that in NS and Scr group. Cytokine levels were determined by ELISA and were presented as mean ± STD (n = 7–8/group). Symbols delineate statistical significance compared to NS mice (*, P < 0.05) and Scr mice (#, P < 0.05). NS: normal saline-treated smoke-induced mice; Decoy: NF-κB decoy ODNs-treated smoke-induced mice; Scr: scrambled ODNs-treated smoke-induced mice.

Airway inflammation was evaluated in the BALF. Total cell and macrophage count in the BALF recovered from Decoy mice were lower than that from NS or Scr smoke-exposed mice (Fig. 4). Moreover, the level of MCP-1 and MIP-1α in lung homogenates was greatly reduced in the decoy group compared with the NS smoke-exposed group (Fig. 5A), and weakly correlated with total cell number (P = 0.051, ρ = 0.619; P = 0.052, ρ = 0.75, respectively). Instillation of NF-κB decoy ODNs induced a significant increase in TNF-α protein levels in mice BALF. In contrast, the level of IL-6 in BALF was not significantly changed(Fig. 5B).

In addition, PIF and PEF were measured to determine whether instillation of NF-κB decoy ODNs influences lung function. As expected, administration of NF-κB decoys but not scrambled ODNs led to a significant improvement of PEF (Table 4).

**Table 4:** Respiratory function in cigarette smoke-exposed mouse groups on day 92.

Treatment	PIF(L/S)	PEF(L/S)
NS	1.46 ± 0.23	3.69 ± 0.45*
Decoy	1.83 ± 0.34	5.46 ± 0.44
Scr	1.62 ± 0.28	3.79 ± 0.21#

Data were expressed as mean ± STD. PIF: peak inspiratory flow PEF: peak expiratory flow. NS: normal saline-treated smoke-exposed mice; Decoy: NF-κB decoy ODNs-treated smoke-exposed mice; Scr: scrambled ODNs-treated smoke-exposed mice. *P < 0.05 for Decoy mice compared with NS mice, # P < 0.05 for Decoy mice compared with Scr mice. n = 8/per group

### NF-κB decoy ODNs treatment induced pro-MMP-9 in BALF, but did not affect pathological changes in small airways and alveoli

**Figure 6:**
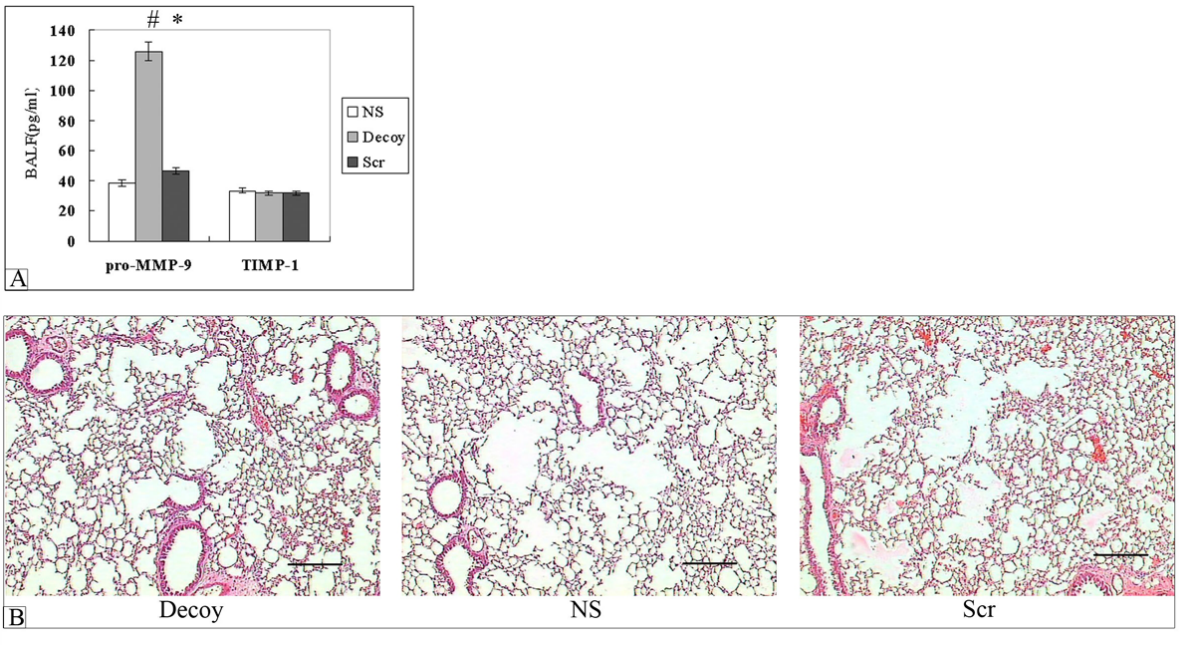
**The effect of administration NF-κB decoy ODNs on the structure of pulmonary parenchyma and the expression of pro-MMP-9 or TIMP-1 in the long-term smoke-induced mice**. (A)NF-κB decoy ODNs significantly induced high levels of pro-MMP-9 but not TIMP-1 in the BALF of mice. Data were expressed as mean ± STD (n = 8). Symbols delineate statistical significance compared to NS mice (*, P < 0.05) and Scr mice (#, P < 0.05). (B) Lung parenchyma from NS, Decoy or Scramble-treated smoke-exposed mice at 92 days. Mice were exposed to smoke for 92 days, then they were killed 1 day after the last exposure and their lungs processed for light microscopy with haematoxylin-eosin staining. The lesion was characterized by disseminated foci of airspace destruction interspersed by apparently normal parenchyma. Original magnification 100×, Bar = 200 μm. NS: normal saline-treated smoke-induced mice; Decoy: decoy NF-κB ODNs-treated smoke-induced mice; Scr: scrambled ODNs-treated smoke-induced mice. n = 8.

The concentration of MMP-9 was undetectable in mouse BALF in our experiment, We therefore measured the levels of pro-MMP-9 and TIMP-1, which have been shown to be tissue remodeling-related. Moreover, NF-κB is a critical transcription factor in the regulation of MMP-9. Of note, NF-κB decoy ODNs not scrambled ODNs modified the levels of pro-MMP-9. Additionally, there was no significant change in the expression of TIMP-1 (Fig. 6A).

We evaluated alveolar wall destruction and enlargement of alveolar spaces by morphologic and morphometric analyses. The level of alveolar wall destruction was determined by measuring the DI and enlargement of alveolar spaces, and by quantifying the Lm and the Am. Microscopic analysis of lung tissue sections revealed clearly the enlarged destroyed alveolar spaces interspersed by apparently normal parenchyma among NS, Decoy and Scr groups (Fig. 6B). Unexpectedly, no significant difference was found in Lm, Am, or DI calculated values after 92 days of smoke exposure (Table 5).

**Table 5:** Lung Morphologic analysis of mice on 92 days of persistent smoke exposure.

Treatment	Lm(μm)	Am(μm^2^)	DI
NS	46.05 ± 6.71	1019.71 ± 95.62	42.89 ± 9.19
Decoy	45.07 ± 8.23	1137.15 ± 246.28	48.73 ± 15.87
Scr	41.48 ± 4.51	1231.02 ± 139.88	44.54 ± 11.70

Pr > Chi-Square	0.4573	0.0643	0.4712

Data were analyzed by Nonparametric Mann-Whitney U-test and expressed as mean ± STD. Lm: linear intercept; Am: mean alveolar surface; DI: destructive index; NS: normal saline-treated smoke-exposed mice; Decoy: decoy NF-κB ODNs-treated smoke-exposed mice; Scr: scrambled ODNs-treated smoke-exposed mice. n = 8

Based on a blinded assessment of the pathology, the examination of small airways post administration NF-κB decoy ODNs revealed fibrosis was prominent after administration NF-κB decoy ODNS in peribronchiolar and interstitial lung tissue compared to treatment with scramble ODNs while goblet-cell metaplasia scores significantly reduced compared to NS lung specimens (Table 6).

**Table 6:** Mean Group Score ± STD for Each Pathological Variable on 92 days of persistent smoke exposure.

Group	Goblet-Cell Metaplasia	Inflammatory-Cell Infiltration	Fibrosis	Muscle
NS	9.17 ± 5.05	5.50 ± 1.48	6.42 ± 2.15	5.58 ± 1.88
Decoy	3.70 ± 2.54*	6.79 ± 4.00	9.71 ± 4.27#	5.08 ± 2.42
Scr	5.30 ± 2.19	4.5 ± 1.58	4.68 ± 2.26	4.94 ± 2.59

Data were analyzed by Nonparametric Mann-Whitney U-test and expressed as mean ± STD. NS: normal saline-treated smoke-exposed mice; Decoy: decoy NF-κB ODNs-treated smoke-exposed mice; Scr: scrambled ODNs-treated smoke-exposed mice. *P < 0.05 for Decoy mice compared with NS mice, # P < 0.05 for Decoy mice compared with Scr mice. n = 5–7/per group

## Discussion

Smoke-induced chronic airway inflammation may be mediated by overwhelming inflammatory dysregulation caused by overexpression of not one or several but many NF-κB regulated genes. We here tested the hypothesis that blockade of NF-κB transcriptional activity, via phosphorothioate-modified decoy ODNs containing the NF-κB consensus binding site, would improve smoke-induced chronic airway inflammation and prevent lung dysfunction in the mouse model system. Our results provided evidence that local administration of decoy through trachea indeed make a strong decrease of a population of macrophages in BALF and alveolar space of smoke-induced mice. Moreover, NF-κB-regulated chemokines MCP-1 and MIP-α were strongly repressed in mice BALF after administration of NF-κB decoy ODNs intratracheally compared with NS-treated smoke-triggered mice. Conversely, NF-κB decoy ODNs increased release of TNF-α and pro-MMP-9 in the mice BALF. These data show that NF-κB decoy ODNs have both repressive and stimulating effects on NF-κB-regulated inflammatory genes in the mouse model.

We report here that intratracheal administration of decoy ODNs, but not scrambled control, abrogated NF-κB activation in whole lung following long-term cigarette exposure; furthermore, we determined that such treatment was very effective in preventing the development of lung dysfunction and macrophage aggregation in the airway.

Systemic or local injection of "naked" NF-κB decoys may effectively inhibit NF-κB activation and thereby prevent inflammation in vivo [25,38]. As reported here, we've demonstrated that intratracheal administration of "naked"NF-κB decoys with modified phosphorothioate backbones resulted in reduced NF-κB activation, while no effect was observed after scrambled ODN administration. The decoy ODNs used in this study were phosphorothioated and therefore resistant to degradation. Although we cannot exclude that the decoy ODNs were damaged through smoke exposure, there is good evidence that 24 h after intravenous injection at least 50% of phosphoratioated ODN in the lung were intact [39]. We have previously shown that NF-κB activation slightly increased compared to air-exposure mice in a model of subacute inflammation [27]. Here, we demonstrate that long-term smoke exposure in mice enhanced NF-κB activity in the nuclear extracts of lung tissue. The success in vivo transfer of a sufficient quantity of NF-κB decoy ODN into lungs was confirmed by the gel shift assay. These results encouraged us to study the potential of NF-κB decoy ODNs for pulmonary smoke-induced chronic airway inflammation by in vivo via intratracheal administration.

### 1. Intratracheal delivery of NF-κB decoy ODNs reduced macrophage influx and prevented lung dysfunction in smoking mice

Many of the genes implicated in smoke-induced chronic airway inflammation contain NF-κB binding sites in the promoter/enhancer region (i.e., cytokines, chemokines and proteases) [1-4]. Of particular clinical relevance, NF-κB binding activity has been reported to increase in smokers and is correlated with lung function [15].

In our study, there was no significant increase in the influx of neutrophils following 92 days smoke exposure, neither in BALF nor lung parenchyma. This result agreed with some previous studies, which also have shown that the inflammatory cell type is cigarette dose-dependent [40] and related with smoking history in COPD patients [41]. Macrophages have a potential role in the pathogenesis of COPD which has several important functions such as phagocytosis[42], activating the adaptive host response[43]. The alveolar macrophage products include cytokines and chemokines with the capacity of recruiting other inflammatory cells to the lungs [44]. Furthermore, there is a positive association between macrophage numbers in the alveolar walls and the presence of mild-to-moderate emphysema as well as the degree in small airways disease in patients with COPD [45]. Nuclear localisation of p65 in CD68^+ ^alveolar macrophages rather than neutrophils confirmed the presence of activated NF-κB in lung parenchyma macrophages of patients with stable COPD [46]. Therefore, we underline the importance of studying NF-κB activity in alveolar macrophages in our research. As expected, the macrophage counts in the BALF were reduced and paradoxically decreased in the alveolar regions as assessed by quantificational analysis. Although cigarette smoke can modify matrix proteins, resulting in macrophage activation and adherence in the alveolar spaces together with decrease on alveolar macrophage population in the BALF [47], we can rule out the effect of cigarette smoke on the population of macrophages by comparing NF-κB decoys group to NS group.

It is now clear that macrophage populations can be distinguished based on their surface antigen expression, and functional activity. One population is termed the "inflammatory" monocyte/macrophage population and preferentially traffic to sites of inflammation [48]. This function difference may explain transfection efficiency was not over 50%.

As a result of NF-κB inhibition in mouse lung, MIP-1α and MCP-1 expression in lung was markedly reduced in the airways of decoy-treated mice as compared to NS-treated controls, whereas there was no significant decrease in scramble group compared with NS-treated controls. This result suggested that the inhibition was from NF-κB decoy but not double stranded oligodeoxynucleotides. Prior studies have identified an important role for CC-chemokines such as MIP-1α in macrophage accumulation in the lungs of smokers with severe airflow limitation [49]. It is reasonable to speculate that reduced macrophage recruitment in the airways and alveolar space may be involved in MIP-1α and MCP-1 attenuation or other chemoattractants are involved in macrophage recruitment in this model.

The release of pro-inflammatory mediators might play an important role in long-term smoke-triggered lung inflammation. NF-κB theoretically regulates the secretion of TNF-α and IL-6. However, our data showed administration of NF-κB decoy ODNs did not alter IL-6 levels in lung BALF when compared with scrambled ODNs. Several explanations may account for these differences. Firstly, both of these pro-inflammatory cytokines are produced by a variety of cells types, including macrophages and epithelial cells. NF-κB decoys might not selectively enter into epithelial cells [25]. In addition, IL-6 release is both NF-κB and IKK (inhibitor of κB kinase) 2-dependent in human pulmonary epithelial cells in vitro [50]. Interestingly, in rat asthma model, IκB kinase-2 inhibitor cause significant dose-related and time-dependent inhibition of TNF-α [51]. This inhibition of κB were not studied in this current study and therefore we can not rule out if any effects of the compound are due to IκB regulation of NF-κB pathways.

With regard to TNF-α production, our results showed higher expression in NF-κB decoy ODNs-treated smoke induced mice compared with NS or Scr-treated control mice. The possible explanations for this result may be the relationship between MCP-1 and the inflammatory mediators [52,53]. In addition, there is a diversity in the mechanisms of NF-κB-regulated inflammatory genes, which could explain the reduction in gene expression selectively for MCP-1 and MIP-1α, but not for TNF-α and IL-6 in response to NF-κB decoy ODNs administration. Interestingly, one of conserved NF-κB binding sites in IL-6 gene contained high-affinity AP-1-binding sites, suggesting that the response of some NF-κB dependent genes may be modified by adjacent transcription factor regulatory sites. However, for TNF-α, AP-1 binding sites did not exist in conserved NF-κB binding sites [54]. More work will be required to understand NF-κB and other transcription factors in our model and their regulation function in target inflammatory genes.

As expected, administration of NF-κB decoy ODNs prevented the development of airway dysfunction in our study. Previous study showed that both alveolar macrophage (Ams) counts and MIP-1α levels in BALF were negatively correlated with FEV (1.0% pred) [55]. This suggested that macrophages play an important role in smoking related airflow obstruction. Consistent with above results, the lower level of MIP-1α in BALF may cause macrophage influx reduced in the airways and lung parenchyma, and alleviate airway limitation following decoy treatment.

### 2. Intratracheal delivery of NF-κB decoy ODNs did not prevent pathological changes in small airways and alveolar space in smoking mice

A crucial pathologic feature of COPD is airway inflammation and remodeling. This process primarily occurs at the level of the small airways, defined as bronchioles that are less than 2 mm in diameter in human being. Niewoehner and colleagues indicated that early structural changes in the small airways developed before the diagnosis of COPD was established [23]. We therefore focused on small airways fibrosis differences among the three groups. However, the results were unexpected. There was striking changes in fibrosis and goblet-cell metaplasia reflecting strong function of NF-κB decoy ODNs for tissue structure abnormality.

The treatment outcomes we obtained can be associated with increased expression of pro-MMP-9 and/or TNF-α expression in BALF after treatment with NF-κB decoy ODNs. The increase on MMP-9 profile seems consistent with fibrosis pathological score in small airways in our study. A similar role of MMP-9 has been reported that transgenic MMP-9 expression induces adult-onset emphysema in mice [56]. Despite TIMP-1 is thought to be important in the airway repair and remodeling processes [57,58] and one of regulators of MMP-9[59], its profile remained unchanged in our study. A plausible explanation for the observed effect in MMP-9 levels in BAL fluid among NF-κB decoy ODNs treatment groups is the presence of multiple transcription factor consensus binding motifs in the MMP-9 promoter, including NF-κB, SP-1, AP-1 and each of their binding sites are indispensable for PMA-induced MMP-9 gene transcription in HeLa cells [60]. Although our data demonstrated that there was unaltered in AP-1 activity that are known to promote MMP expression and a combination of supershift, RNA interference and overexpression experiments implicated AP-1 family member Fra-1 in the regulation of MMP-1 expression. It is unclear which transcription factor plays a central regulatory role in MMP-9 expression *in vivo *post cigarette exposure, or whether multiple transcription factors lead to a coordinated response of MMP-9 expression [61]. Gene transcription is also reliant on the modification of core histone proteins, which regulate genome accessibility to transcription factors and cofactors [62].

MMPs are both effectors and regulators of inflammation. Pro-inflammatory stimuli such as TNF-α and IL-1β also increased MMP-9 production in human monocytes [63]while there were examples of MMP-mediated release or activation of cytokines including TNF-α [64]. Together, this suggests the intersection between the chemokine and MMP networks is broad with potentially important biological consequences. In our results, we observed the elevation of pro-MMP-9 profile in concomitance with higher level of TNF-α and lower expression of MCP-1 and MIP-1α post treatment with NF-κB decoy ODNs. It is possible that cytokines and MMPs networks play a key role in orchestrating the inflammation via non-dependent NF-κB pathway in smoke-induced mice model. Other studies have documented possible effects of TNF-α on the development of pulmonary fibrosis through chronic lung inflammation and activation of the elastolytic enzymes [65]. In particular, NF-κB site is indispensable for the suppressive activity of TGF-β in the regulation of MMP-9 transcription[66].

## Conclusion

We reported here that local NF-κB inhibition was associated with attenuated MIP-1α and MCP-1 expression simultaneously, macrophage influx in the airway and lung parenchyma, and marked improvement in respiratory function of mice in response to long-term smoke exposure. Our studies suggest that inhibitors of NF-κB may offer promise as a therapeutic approach for the improvement of smoke-triggered pulmonary dysfunction. Furthermore, our pathological analysis of the macrophages reduction in lungs of mice and macrophages recruitment-related cytokines decrease also provides useful information about NF-κB decoy ODNs for a model of experimental smoke-induced chronic inflammation.

## Competing interests

The authors declare that they have no competing interests.

## Authors' contributions

YTL participated in the design of the study, carried out the immunoassays and cytologic studies, performed the statistical analysis and drafted the manuscript. BH supervised the design of the study, participated in the statistical analysis and coordination. YZW participated in the morphometric analysis. JW participated in the morphometric analysis.
